# Clinicopathologic Correlation of Large Gastric Polyps in an Elderly Female: Case Report and Review of the Literature

**DOI:** 10.7759/cureus.52806

**Published:** 2024-01-23

**Authors:** Abdulaziz S Taleb, Babatope L Awosusi

**Affiliations:** 1 Gastroenterology and Hepatology, King Khalid Hospital, Al Majma'ah, SAU; 2 Pathology, King Khalid Hospital, Al Majma'ah, SAU

**Keywords:** endoscopy, gastritis, helicobacter pylori, fundic gland polyp, gastric

## Abstract

Fundic gland polyps (FGPs) are benign epithelial polyps usually located in the gastric body and fundus. Here, we describe the case of an elderly woman who presented with symptomatic anemia, abdominal pain, and weight loss. There was also history of chronic use of proton pump inhibitors for the symptomatic treatment of dyspepsia. Reflux esophagitis, duodenal ulcers, and multiple gastric polyps suspicious of malignancy were found at endoscopy. A large pedunculated antral polyp measuring more than 10mm in the largest dimension and other smaller polyps in the body and fundus were removed completely by cold snare and sent for histopathologic evaluation. The polyps were eventually diagnosed as benign fundic gland polyps. Biopsies taken from the gastric mucosa also showed Helicobacter pylori-associated gastritis. Helicobacter pylori eradication therapy was commenced with the resultant resolution of symptoms. No surveillance is required for FGPs because they are not premalignant lesions. However, large FGPs require excision and thorough histopathologic evaluation to rule out atypia and malignancy. This index case further highlights a possible causal link between the chronic use of proton pump inhibitors and the development of fundic gland polyps.

## Introduction

Fundic gland polyps (FGPs) are benign polyps usually located in the gastric body and fundus [1]. The incidence of FGPs has increased in recent years; they have become the most common type of gastric polyp [1]. The histo-morphologic features of FGPs include fundic glands that are cystically dilated and lined by parietal cells, chief cells, and mucous neck cells [1]. 

FGP can be either sporadic or syndromic [2,3]. Sporadic polyps are usually less than ten, while in syndromic cases, the number of polyps is many, with associated dysplasia and risk of malignant transformation in 50 to 100% of cases [3]. The polyps are usually biopsied or resected at endoscopy and submitted for histopathologic evaluation, especially the large ones that are more than 10mm in widest dimension [3]. The reported prevalence of FGPs seen in routine upper gastrointestinal endoscopy ranges from 0.5% to 11.1%, while the recent increase in incidence has been attributed to the chronic or prolonged use of proton pump inhibitors (PPIs) [1,3]. 

Here, we describe the case of an elderly woman who presented with symptomatic anemia, abdominal pain, and weight loss. At endoscopy, multiple gastric polyps suspicious of malignancy were found. The polyps were eventually diagnosed as benign fundic gland polyps after histopathologic evaluation.

## Case presentation

We hereby present the case of a 74-year-old female who was seen at the emergency department with a history of dizziness, shortness of breath, physical exertion, abdominal pain, and weight loss. There is no past medical history of chronic illnesses or malignancy. The patient also reported a history of chronic use of proton pump inhibitors for symptomatic treatment of dyspepsia.

On examination at presentation, the abdomen was soft and lax with mild tenderness on deep palpation of the right upper quadrant, the epigastrium, and peri-umbilical regions.

Initial laboratory work-up showed anemia with a hemoglobin value of 5.8g/dl. Electrolytes, serum urea, and creatinine values were within the normal range. Liver function test results were also within the normal reference range. An abdominal ultrasound revealed small ascites.

**Figure 1:**
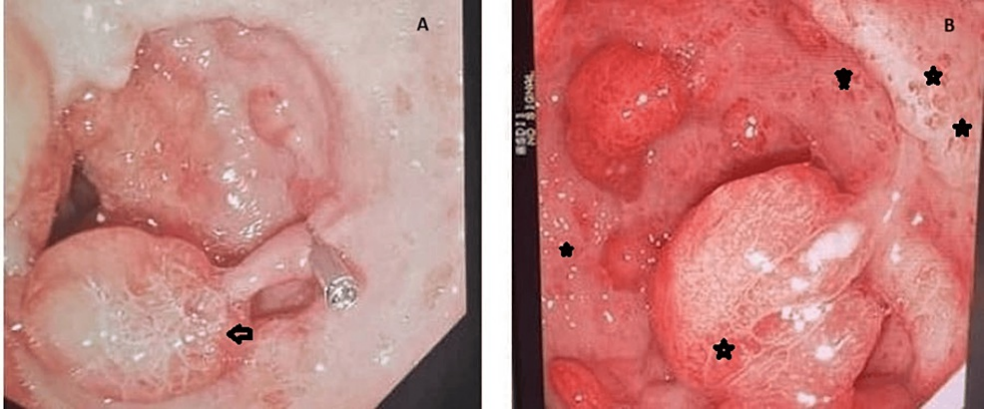
Upper gastrointestinal endoscopic pictures Figure 1A: Shows a large pedunculated polyp in the gastric antrum (black arrowhead) Figure 1B: Shows gastric mucosa with areas of punctate hemorrhages (black stars)

She underwent an initial upper gastrointestinal (GI) endoscopy that showed reflux esophagitis (Los Angeles grade B) and scattered polypoid masses suggestive of malignancy. A large pedunculated polyp was seen in the gastric antrum with areas of punctate hemorrhages in the surrounding mucosa (Figure 1A, 1B). A Forrest class III ulcer was also seen in the first part of the duodenum. Multiple gastric biopsies were taken that showed features consistent with Helicobacter pylori-associated gastritis, and she was commenced on a high-dose proton pump inhibitor (omeprazole).

Two weeks later, a second upper GI endoscopy showed an increase in the size and number of gastric masses. The large pedunculated antral polyp seen was removed by cold snare and sent for histopathologic evaluation. A healed duodenal ulcer was seen in the first part of the duodenum.

Helicobacter pylori eradication therapy was commenced, comprising amoxicillin, clarithromycin, levofloxacin, and omeprazole (20 mg PO BID) for 14 days. Subsequently, the patient improved clinically; the abdominal pain, dizziness, and shortness of breath resolved, and the hemoglobin level also increased to 12 g/dl after three months of treatment with haematinics.

**Figure 2:**
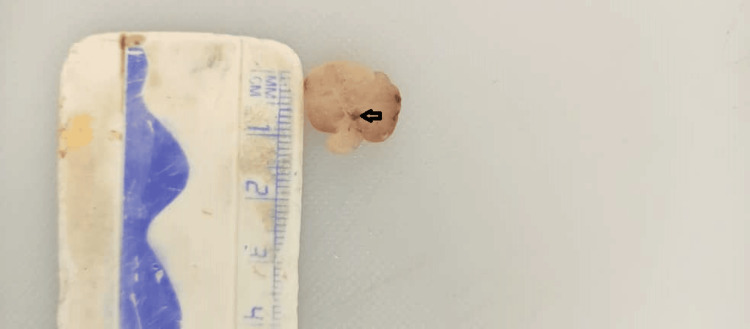
Figure shows the cut surface of the large pedunculated gastric polyp with a small cyst cavity (black arrow)

**Figure 3:**
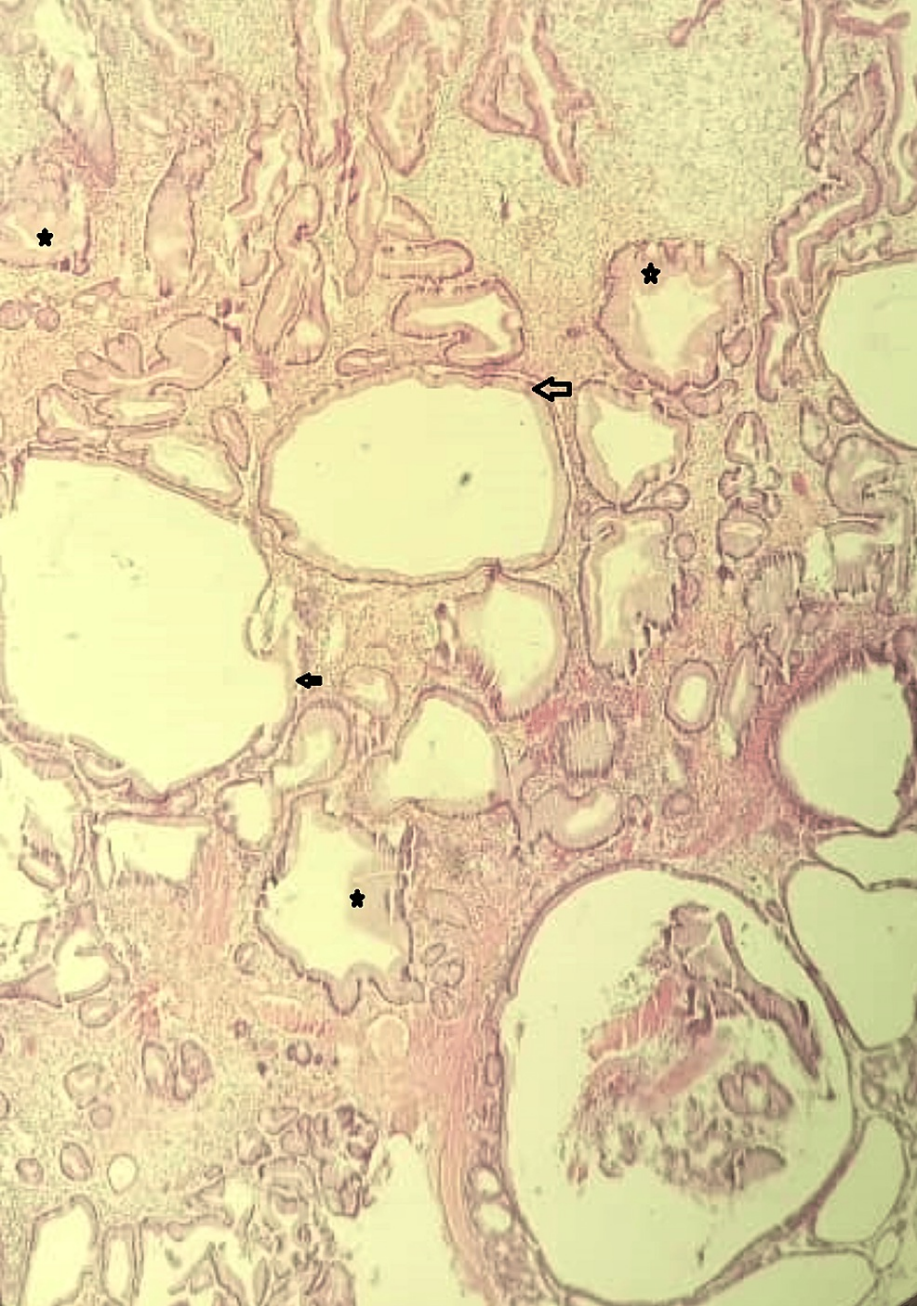
Figure shows a fundic gland polyp with cystically dilated glands (black arrows) and hyperplastic parietal cells (black stars). Hematoxylin and eosin stains x400 magnification

The specimen received at histopathologic grossing was a soft grey-white polyp weighing 1.2g and measuring 1.5x1cm (Figure 2), with 10 smaller pieces of soft grey-white tissues measuring 1x0.5cm in aggregate dimension. The microscopic finding shows that a section of the gastric polypectomy specimen features consistent with fundic gland polyp (Figure 3).

Four months later, a follow-up upper GI endoscopy showed a reduction in the size and number of the gastric polyps, but the patient was still positive for Helicobacter pylori. The treatment plan was changed to Pylera (Bismuth Sub-citrate potassium, metronidazole, and tetracycline). The patient then improved significantly and is being followed up at the medical outpatient clinic.

## Discussion

Fundic gland polyps (FGPs) account for 16 to 51% of all gastric epithelial polyps and are the most frequently seen benign gastric polyps [4,5]. FGPs were initially thought to be syndromic extra-colonic manifestations of Familial adenomatous polyposis (FAP) because they have been seen in association with both FAP and attenuated FAP [6]. Other disease associations include Gardner syndrome and Zollinger-Ellison syndrome [6].

Sporadic FGPs are predominantly seen in females in their seventh to eighth decades. In contrast, syndromic FGPs occur earlier during the fourth and fifth decades, with similar incidence in both men and women [6]. In this index case, it affected a 72-year-old elderly female, which is similar to the reported epidemiology. Genetic studies have described a relationship between FGPs and somatic mutations in the β-catenin gene [7]. Though the presence of these β-catenin gene mutations in FGPs indicates a neoplastic nature, it is proven that FGPs have very low malignant potential [7].

The etiopathogenetic link between PPIs and FGPs has been controversial [6]. Graham was the first person to describe the probable causal role of PPI use in FGP formation [6]. There is still no definitive mechanism that explains the pathogenesis of FGPs secondary to the use of PPI; the proposed pathway for polyp formation is thought to be due to hypergastrinemia and gastric mucosal hypertrophy [6]. Sporadic FGPs are seen predominantly in individuals who undergo upper gastrointestinal endoscopy for symptoms of gastroesophageal reflux disease and heartburn [6]. A nine-year study by Declich and colleagues on seventy patients with sporadic FGPs showed that 34% of these patients had esophageal complaints, GERD, and hiatal hernia [7]. The patient presented in this index case also had endoscopically confirmed reflux esophagitis and duodenal ulcers apart from the large fundic gland polyps seen.

Bertoni et al. reported that when PPI is used for over one year, there is a fourfold increased risk of the development of FGP [7]. This index patient has a history of use of PPI for dyspeptic symptoms, but the specific duration could not be confirmed with certainty. Therefore, our case was considered sporadic and probably PPI-related. A report from Japan stated that after rabeprazole was used for 104 weeks, 13.6% of patients developed new fundic gland polyps, predominantly in the gastric corpus, with sizes less than 5 mm [8]. In this index case, though there were multiple polyps in the fundus and body, a large antral polyp measuring 15 × 10 × 10 mm was seen, larger than the usual FGP. This increased the clinical suspicion of gastric malignancy in association with the clinical symptom of weight loss and laboratory confirmation of anemia in this elderly patient. The large antral polyp was resected completely; some of the other polyps in the body and fundus were also biopsied and sent for histopathologic evaluation.

Some fundic gland polyps can occasionally cause symptoms of bleeding, anemia, or gastric outlet obstruction [9]. In this index case, the patient presented with symptoms of anemia, weight loss, and abdominal pain. The definitive diagnosis of fundic gland polyp was made after histopathologic evaluation with evidence of Helicobacter pylori infection and gastritis. No focus of atypia or malignant change was seen in any of the polyps and mucosal biopsies evaluated. The specimen in this case demonstrated fundic gland hyperplasia and parietal cell protrusion, which has been described in patients with long-term PPI use [9]. The morphologic changes that have been attributed to long-term PPI therapy include hyperplasia of parietal cells, stromal edema, and cystic dilatation of the fundic gland ducts [9].

It has been noted that Helicobacter pylori infection can cause regression of FGPs with subsequent enlargement of the remaining polyps after Helicobacter pylori eradication [6]. This is also similar to what happened in this index case; the initial follow-up endoscopy that was done after Helicobacter pylori eradication treatment showed an enlargement of the FGPs. A repeat follow-up endoscopy done four months later showed a reduction in the size and number of the polyps with clinical resolution of symptoms.

No surveillance is required for FGPs because they are not premalignant lesions; Genta and colleagues found no increase in gastric neoplasia in patients with FGPs [10].

## Conclusions

This index case further highlights a possible causal link between the chronic use of proton pump inhibitors and the development of fundic gland polyps. Many patients use PPIs for prolonged periods in the treatment of gastroesophageal reflux disease and other related conditions without being aware of this potential adverse effect. PPIs should be prescribed only when necessary, and the duration of use should be carefully regulated. Large fundic gland polyps require thorough histopathologic evaluation to rule out atypia and malignancy.
